# Characteristic rotational behaviors of rod-shaped cargo revealed by automated five-dimensional single particle tracking

**DOI:** 10.1038/s41467-017-01001-9

**Published:** 2017-10-12

**Authors:** Kuangcai Chen, Yan Gu, Wei Sun, Bin Dong, Gufeng Wang, Xinxin Fan, Tian Xia, Ning Fang

**Affiliations:** 1 Department of Chemistry, Georgia State University, Atlanta, GA 30303 USA; 2 Department of Chemistry, Iowa State University, Ames, IA 50011 USA; 3 Department of Electronics and Information Engineering, Huazhong University of Science and Technology, Wuhan, 430074 China; 4 Present Address: The Bristol-Myers Squibb Company, Devens, MA 01434 USA; 5 Present Address: Corning Inc., Painted Post, NY 14870 USA; 6 Present Address: Department of Chemistry, North Carolina State University, Rayleigh, NC 27695 USA

## Abstract

We report an automated single particle tracking technique for tracking the *x*, *y*, *z* coordinates, azimuthal and elevation angles of anisotropic plasmonic gold nanorod probes in live cells. These five spatial coordinates are collectively referred to as 5D. This method overcomes a long-standing challenge in distinguishing rotational motions from translational motions in the *z*-axis in differential interference contrast microscopy to result in full disclosure of nanoscale motions with high accuracy. Transferrin-coated endocytic gold nanorod cargoes initially undergo active rotational diffusion and display characteristic rotational motions on the membrane. Then as the cargoes being enclosed in clathrin-coated pits, they slow down the active rotation and experience a quiet period before they restore active rotational diffusion after fission and eventually being transported away from the original entry spots. Finally, the 3D trajectories and the accompanying rotational motions of the cargoes are resolved accurately to render the intracellular transport process in live cells.

## Abstract (web summary)

Distinguishing rotational motions from translational motions in the *z*-axis has been a long-standing challenge. Here the authors develop a five-dimensional single particle tracking method to detect rotational behaviors of nanocargos during clathrin-mediated endocytosis and intracellular transport.

## Introduction

**Fig. 1:**
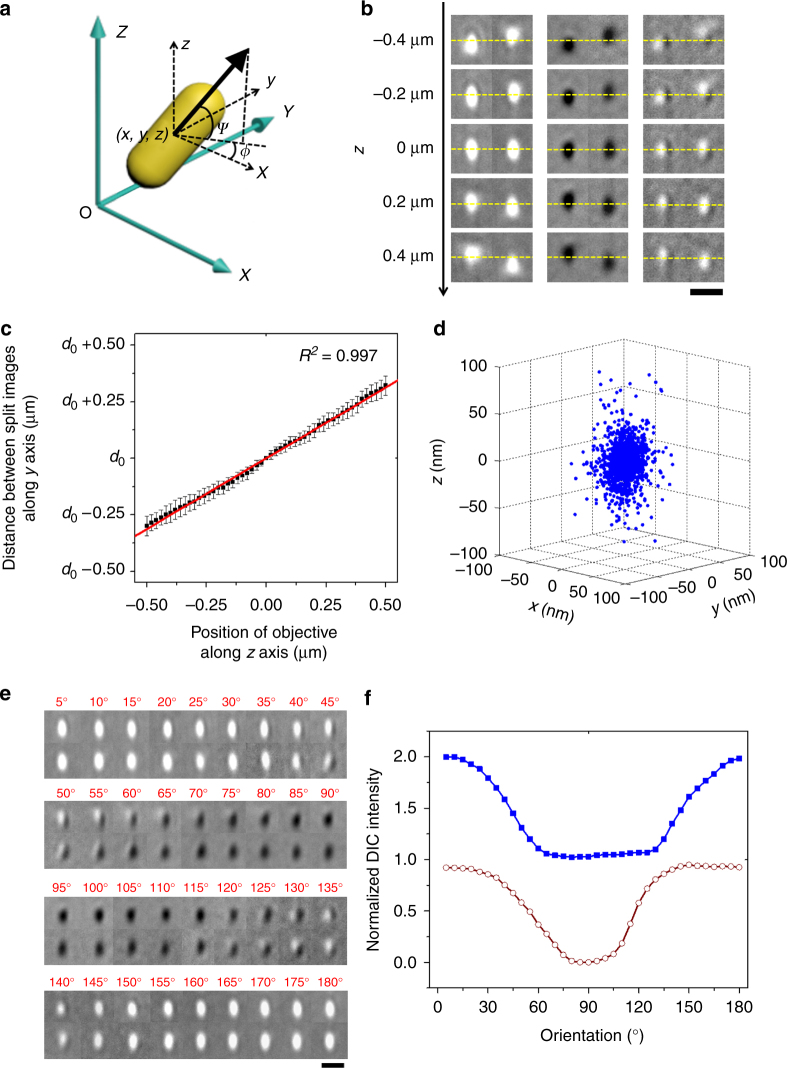
Principle and localization precision of the parallax-DIC microscope. **a** Schematic illustration of a gold nanorod placed in the 3D coordinate system. The *x*- and *y*-axes are set according to the polarization directions of the illumination light. The dipole shown here corresponds to the long axis of the gold nanorod, whose centroid locates at (*x*, *y*, *z*). The azimuthal angle and the elevation angle of the longitudinal axis of the gold nanorod are presented as *ϕ* and *ψ*, respectively. **b** Bright (left), dark (middle), and half-bright/half-dark (right) images of a gold nanorod (40 × 80 nm) at different orientations and vertical positions. The half-plane images are aligned by the nanorod’s center of mass at the focal plane (*z* = 0 μm). The yellow dashed lines indicate the *z*-position of the center of the gold nanorod in focus. Scale bar is 1 μm. **c** Calibration of the 5D-SPT technique. The distance between the two half-plane images changes linearly with the *z*-position of the gold nanorod within ±0.5 μm of the focal plane. The distance for an in-focus gold nanorod is defined as *d*_0_. The error bars reflect the standard deviations of the distances measured when the nanorod’s azimuthal angle *ϕ* changes in the range of 0–180° with 5° steps (error bar ± s.d., *n* = 36). The calibration curve returned a slope of +0.626 (*d*/Δ*z*) and *R* ^2^ of 0.997. **d** Three-dimensional localization distribution of the gold nanorod with standard deviations of 11 nm in *x*, 14 nm in *y*, and 17 nm in *z*. **e** The in-focus half-plane image patterns of a gold nanorod at different azimuthal angles when the sample slide is rotated in 5° steps for 180°. The scale bar represents 1 μm. **f** The normalized bright part (blue) and dark part (brown) intensities of the half-plane images on the top in each pair in E

Single particle tracking (SPT) has become an indispensable approach in our attempts to understand the detailed working mechanisms of biomolecules in complex cellular environments^1^. Motions of single imaging probes recorded in SPT experiments contain rich information, including the *x*, *y*, *z* coordinates and the two orientation angles (azimuthal angle *ϕ* and elevation angle *ψ*, as defined in Fig. 1a) of the probe’s transition dipole over a series of time steps. After two decades of development, lateral localization of single imaging probes with nanometer accuracy^2–4^ in the (*x*, *y*) plane has become routine, and many strategies have also been developed to improve the *z*-tracking ability^5–18^. However, the ability to acquire accurate measurements in all five spatial parameters (*x*, *y*, *z*, *ϕ*, *ψ*) simultaneously remains elusive. These five coordinates are collectively referred to as five dimensions (5D) in the present study.

The challenges are mainly two-fold. First, it is difficult to resolve the dipole orientation of single fluorescent probes in a cellular environment with reasonably fast temporal resolution due to the well-known limitations, such as high background and fast photobleaching. This challenge can be circumvented by using anisotropic plasmonic gold nanorods as alternative rotational tracking probes. The exceptionally high absorption and scattering cross-sections of gold nanorods result in strong and stable signals at the localized surface plasmon resonance wavelengths^19^. We have previously developed the single particle orientation and rotational tracking (SPORT) technique for direct visualization of rotational dynamics of gold nanorods in differential interference contrast (DIC) microscopy at millisecond temporal resolution, while still obtaining high-contrast images of cellular features^20–23^. Relatively low illumination light intensity from a typical halogen lamp, instead of a stronger laser light source^24^ brings minimal disruptions to cell functions, making it particularly suitable for long-term SPT experiments in live cells. Polarized or defocused dark field (DF) microscopy^25–27^ is another viable choice for tracking single plasmonic gold nanoparticles, although its application to SPT in live cells is more challenging due to the generally high-scattering background in the cellular environments. In addition, the two-photon luminescence^28^ and photothermal heterodyne imaging^29^ can also image gold nanorods; however, the relatively high-power laser light source and generally slow raster-scanning process can limit their applicability in single cell imaging. Furthermore, significant progresses have also been made in measuring gold nanorods in vivo^30–32^; however, these in vivo methods were not suitable for disclosing the motions of individual gold nanorods due to insufficient sensitivity or speed.

The second challenge is associated with the difficulties to distinguish rotational motions of the anisotropic probe from its movement in the *z*-axis because both types of motion result in similar changes in signal in DIC microscopy. This ambiguity would result in incorrect assignment of the dipole’s orientation.

Herein we introduce an automated five-dimensional single particle tracking (5D-SPT) technique to overcome these challenges. The core idea is to combine the DIC microscopy-based SPORT technique with the principle of parallax microscopy^15, 16^ for simultaneous spatial and rotational tracking. Parallax is a mechanism for humans to gain depth perception by creating a displacement in the apparent position of an object viewed in two eyes. It is worth noting that the dependence of the defocused DF image patterns on the probe’s relative axial distance to the focal plane can be utilized for axial localization^28, 33^; however, the complexity of the defocused image patterns and relatively weak defocused signal intensities lead to low axial localization accuracy and precision, as well as small vertical tracking distance.

The 5D-SPT technique has been applied to disclose the characteristic motions of transferrin-coated gold nanorod cargos at each stage of the clathrin-mediated endocytosis (CME) pathway and subsequent intracellular transport. This technique makes significant improvements in our ability to detect the presence, location, orientation, translation, and rotation of nanoparticle probes in live cells. Other worth noting advantages include the simultaneous visualization of cellular features with high contrast and the ability to follow target probes for long durations (limited mainly by cell viability and computer data acquisition) and in large 3D volumes (within the camera’s field of view and the travel distance of a high-precision piezo objective scanner). Using the 5D-SPT technique, the current study focuses full motions of cargos in live cells have been resolved with unprecedented details and accuracy.

## Results

### Design of parallax-DIC microscopy

The SPORT technique previously developed in our laboratory takes the advantage of the plasmon resonance-associated birefringence of gold nanorods^25, 27, 29, 34^ and Nomarski-type DIC microscopy^35, 36^, which utilizes two orthogonally polarized beams for illumination. Optically anisotropic gold nanorods show as diffraction-limited spots with bright and dark peaks on a gray background in DIC images (Supplementary Fig. 1). The bright and dark intensities are independent measures of the effective projections of the nanorods onto the two polarization axes, from which the 3D orientation of gold nanorods can be resolved in each DIC image^20, 22, 23^. The normalized DIC bright and dark intensities traces can be readily converted to the orientation measurements.

The new 5D-SPT technique uses parallax to sense the axial movement of the target object, and at the same time, employs an automatic feedback loop algorithm to control the focal plane of the objective and bring the target object back to focus repetitively. In such an implementation, the target object is kept in focus to provide the highest possible S/N for high-accuracy 2D localization and orientation determination, while the axial (*z*) positions are recorded as the positions of the high-precision objective scanner.

**Fig. 2:**
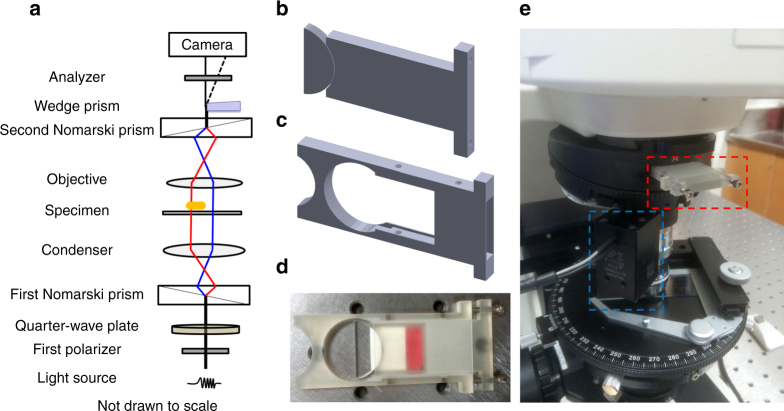
Parallax-DIC microscope and 5D-SPT instrumental set-up. **a** Schematic illustration of the optical path in parallax-DIC microscope. High-precision objective scanner was not drawn in the scheme. **b** Model of the insert mounted with the custom-cut wedge prism. **c** Model of the slot. **d** Photograph of the wedge prism set installed on the 3D-printed insert housing in the 3D-printed slot. The position of the insert and wedge prism were optimized and fixed by four #4/40 × 1/8 socket set screws and two #4/40 × 1/2 hex socket head cap screws to split the light in half at the back focal plane of the objective. **e** Photograph of the 5D-SPT instrumental set-up. The optimized wedge prism set was insert to the light path (as indicated in the red dashed box) to enable the parallax-DIC mode and the objective scanner (as shown in the blue dashed box) was use to deploy the automated 5D-SPT

Parallax is realized on an upright DIC microscope by inserting a wedge prism at the back focal plane of the objective (Fig. 2). Half of the light that has passed through the sample maintains the original path, while the other half of the light is deviated slightly by the wedge prism. Two images of the same sample are formed in the upper and lower halves of the sensor area. These images will be referred to as “half-plane” images in the rest of the discussion because each image utilizes half of the numerical aperture of the objective, while conventional microscopy images utilizing the full numerical aperture will be referred to as “full-plane” images.

The movement of a target object in the axial (*z*) direction is resolved by monitoring the distance (*d*) between the pair of half-plane images of the same object. For any object in the focal plane, the distance between its two half-plane images (*d*_0_) is a fixed value determined by the deviation angle of the wedge prism. When the target object moves upwards (Δ*z* > 0) towards the objective, the two half-plane images move away from one another to increase *d*. When the target object moves downwards (Δ*z* < 0) away from the objective, the two images move toward one another to shorten *d*. Supplementary Movie 1 shows parallax-DIC images of a stationary gold nanorod at three orientations traveling smoothly from −0.4 μm below the focal plane to +0.4 μm above the focal plane at a speed of 100 nm per second with a single frame exposure time of 200 ms. The nanorod shows orientation-dependent DIC images (Fig. 1b).

The parallax-DIC microscope inherits the conventional DIC microscope’s ability of visualizing the cell morphology and detailed intracellular structures. Supplementary Fig. 2 shows the two half-plane images of a live cell at different vertical sections.

### Localization in parallax-DIC microscopy

Localization in parallax-DIC microscopy is essentially a task of finding the accurate positions of the pair of half-plane images. Gaussian fitting, the most widely used localization method, is not suitable for DIC microscopy because the anti-symmetric DIC point spread function (PSF), which consists of apposed bright and dark portions over a gray background, cannot be fitted with a simple mathematical function. Therefore, the more complicated model-based correlation mapping procedures are required for localizing particle probes in DIC microscopy^37, 38^. Localization in parallax-DIC microscopy is even more challenging as the half-plane PSF appears to be stretched in the direction perpendicular to the split plane, as illustrated in the simulated “half-plane” images (Supplementary Fig. 3).

To overcome the complications in the parallax-DIC PSF, we have implemented a redesigned correlation mapping procedure, in which one of the half-plane images is used as the model to map the other image in order to find the centers of these two images. With the wedge prism splitting the light in half, the two resulting half-plane PSFs appear to be the mirror image of each other. The correlation maps of the two half-plane images with correlation scores above a set threshold (typically 0.4–0.6) are weighed to find their relative coordinates. The distance between the two half-plane images is thus determined and can be translated into the axial position of the target probe. The axial localization is calibrated by scanning the focal plane of the objective vertically in 20 nm steps through a stationary gold nanorod and correlating the distance between the two images with the axial position of the sample (Fig. 1c).

### Evaluation of 5D-SPT

The implementation (Fig. 2) and evaluation of the 5D-SPT technique are discussed in detail in the Supplementary Note 1. The figures of merit are summarized in this section.

It is important to strike an ideal balance among temporal, spatial, and angular resolution in 5D-SPT experiments. These three types of resolving power are competing for the same pool of photons. As the temporal resolution increases, the noise level in the measured bright and dark DIC intensities increases, resulting in less accurate assessment of rotation rate and mode.

The response time of the objective scanner and the speed of its interface to the computer determine the fastest temporal resolution of 5D-SPT technique to be ~1 ms. A series of parallax-DIC images taken at different exposure times are displayed in Supplementary Fig. 4. To ensure sufficiently high-angular resolution and localization precision in live cells, the 40 × 80 nm gold nanorod sample and the exposure time of 33 ms were employed in most of the 5D-SPT experiments reported in this paper, unless otherwise noted. The precision measurements of the 3D localization yield standard deviations of 11, 14, and 17 nm in the *x*, *y*, and *z* axes, respectively (Fig. 1d and Supplementary Fig. 5). The evaluation of the autofocusing under the parallax-DIC microscope is shown in Supplementary Fig. 6.

Finally, a set of parallax-DIC images of in-focus gold nanorod at different azimuthal angles is displayed in Fig. 1e. The bright and dark DIC intensities from each image are plot against the azimuthal angle in Fig. 1f. The orientation-dependent DIC intensities allow the orientation measurements.

### Tracking endocytic nanocargos in 5D-SPT

Using the 5D-SPT technique, the rotational motions of transferrin-coated gold nanorods on the cell membrane and during the subsequent endocytosis by A549 human lung cancer cells were continuously followed. Transferrin^39^ molecules were conjugated to gold nanorod surface through PEG linker molecules to facilitate the cellular uptake^21^. Previous studies showed that CME plays a key role in cellular uptake of transferrin-coated gold nanospheres and nanorods of ~50 nm in size^40^. In the present study, we visualized the dynamic internalization of transferrin-coated gold nanorods by transfected A549 human lung cancer cells to express enhanced yellow fluorescent protein (EYFP)-tagged clathrin^41^. The co-localization of gold nanorods and fluorescent bright spots from EYFP-clathrin gives direct evidence that CME is involved in the internalization of transferrin-coated gold nanorods. Supplementary Fig. 7 shows such a case where three out of eight transferrin-coated gold nanorods binding to the membrane of a transfected A549 cell display co-localized EYFP fluorescence after a 30-min incubation in the cell culture medium containing the colloidal gold nanorods.

In addition, we observed that the internalization of transferrin-coated gold nanorods was strongly dependent on the ambient temperature and osmotic pressure. After incubation at 4 °C for 2 h, a significant amount of gold nanorods were observed binding to the cell membrane, but not being endocytosed. After the temperature was raised to 37 °C, over half of the nanorods initially spotted on the cell membrane were internalized within the first 2 h. In another control experiment, when the cells were treated with a hypertonic media (Sucrose) at 37 °C, significant decreases in the uptake of the nanorods within the first 2 h were observed, compared to the nanorod uptake by the cells not treated with the hypertonic media (Supplementary Fig. 8). The temperature dependence and the response to the hypertonic treatment, which disrupts the formation of clathrin-coated vesicles, are consistent with the previous reports that CME is the key pathway for the cellular uptake of nanometer-sized particles^40, 42, 43^.

Furthermore, specific mean to inhibit clathrin-mediated endocytosis was carried out by siRNA knockdown of AP-2 µ2 subunit in A549 cells. The siRNA oligonucleotide sequence 5′-GAGCAUGUGCACGCUGGCCA-3′ targeting μ2-2 subunit of AP-2 is highly effective to achieve nearly complete depletion of AP-2 with two cycles of transfection, as reported by Motley et al.^44^ and confirmed by others^45, 46^. The siRNA treatment was performed following the established protocol. As shown in Supplementary Fig. 9, control cells showed much higher nanorod uptake compare to the cells subjected to AP-2 depletion.

In all the endocytosis 5D-SPT experiments, the parallax-DIC mode was activated intermittently (every few seconds) by inserting the wedge prism into the light path to compensate for focus drifting and keep the target gold nanorod in focus. During the remaining imaging time, the original DIC and fluorescence modes were operated alternately to acquire the orientation information of gold nanorods and the formation of fluorescent EYFP-clathrin coats.

### Rotational pattern before endocytosis

**Fig. 3:**
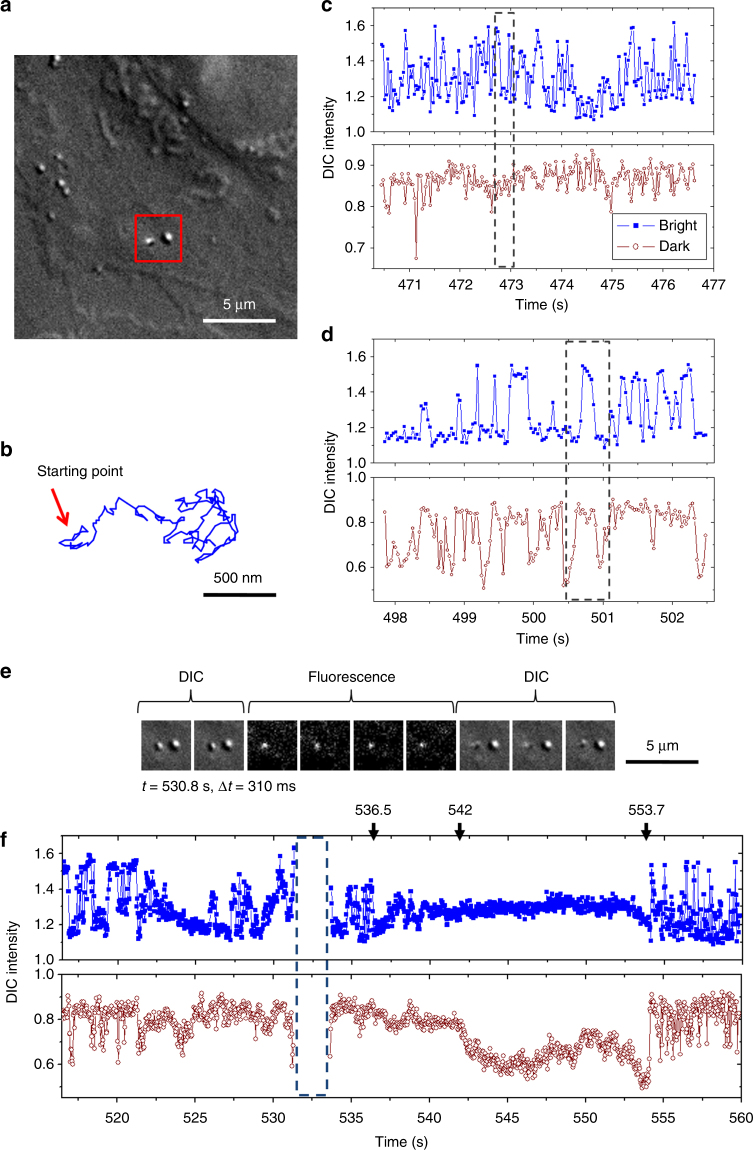
A complete CME event of a gold nanorod by an A549 cell. Corresponding video are presented in Supplementary Movies 1–6. Time 0 is set to the moment when the nanorod first appeared on the membrane. **a** The cell and the location of the gold nanorod probe on the cell membrane. The nanorod (on the left in the red box) was beside a cell feature (on the right in the red box) that showed a constant contrast throughout the recording. **b** The trace of the nanorod’s active translational movement at an early binding stage (Supplementary Movie 2). **c** DIC intensities of the nanorod in the same period as **b**. The framed period shows typical out-of-plane rotations. **d** DIC intensities of the nanorod at a late stage (Supplementary Movie 3). The framed period shows typical in-plane rotations. **e** Sequential DIC and fluorescence images captured in tandem showing that the gold nanorod co-localized with a CCP (Supplementary Movie 4). The movie was acquired at 32 frames per second continuously and the displayed fluorescence images were the average of 10 consecutive images from the movie. **f** DIC intensities of the nanorod before and after scission of the nanorod-containing vesicle (Supplementary Movie 5). The blank period from 531.5 to 532.8 s was toggled to fluorescence mode in checking clathrin fluorescence. The nanorod lost active rotation at 536.5 s and regained rotational and translational freedoms at 553.7 s. The same particle was observed to lose co-localization of fluorescence from EYFP-clathrin, indicating the clathrin disassembly (Supplementary Movie 6). Supplementary Movie 7 shows that the particle was actively transported at 45.7 s after scission in DIC mode

We followed the dynamic uptake of each transferrin-coated gold nanorod continuously from its abrupt appearance on the cell membrane until the nanorod was linearly transported over long distances—convincing evidence of the nanorod being inside the cell. Unlike conventional SPT probes, gold nanorods are easy to identify, giving flickering images due to their own fast rotation on the cell membrane. More importantly, the nanorods display characteristic rotational motions at different binding and endocytic stages. Figure 3 shows such an example of a complete process from the nanorod landing on the cell membrane to being endocytosed by an A549 cell.

When the nanorod first fell on the cell membrane, it showed active lateral diffusion and fast rotation as revealed by the fluctuation of the DIC intensities (e.g., Fig. 3a–d and Supplementary Movies 2 and 3). The rotational motions of gold nanorods can be correlated to the recorded DIC intensity changes as discussed in details in our previous publications based on the correlation of computer-simulated results and experimental data^20, 23^. There are two fundamental rotation modes: the in-plane rotation in which the rotation axis falls in the focal plane and the nanorod rotates within the plane, and the out-of-plane rotation in which the rotation axis is perpendicular to the focal plane and the nanorod rotates out of the plane. Distinctive changing DIC image patterns are associated with these two modes, with a correlated bright and dark intensity changes for the in-plane rotation mode and an anti-correlated bright and dark intensity changes for the out-of-plane rotation mode.

The motions of nanorod at the early stage of binding were characterized by active lateral diffusion in a relatively large area on the cell membrane (e.g., Fig. 3b, c and Supplementary Movie 2) and a random mix of the in-plane and out-of-plane rotation modes as revealed by the DIC intensity changing patterns. For example, during the framed period (472.7–473.2 s) in Fig. 3c, the anti-correlated bright and dark intensity changes suggested that the nanorod likely swung around one of the polarization directions in an out-of-plane rotation mode.

The nanorod gradually lost its translational and rotational freedoms on the cell membrane. The lateral movement quickly became restrained and came to a complete stop at 497.9 s. The rotational motion also became much slower, giving much less frequent fluctuations in the DIC intensities (Fig. 3d and Supplementary Movie 3). From the DIC intensities, we can identify that a component of in-plane rotation mode became more pronounced. For example, during the framed period (500.4–501.1 s) in Fig. 3d, the nanorod’s DIC intensities changed in a correlation manner from completely dark to completely bright and then to completely dark again, showing a typical in-plane rotation mode.

The gradual loss of translational and rotational freedoms and the increase of the in-plane rotation component were an indication of the assembling of the endocytic machinery around the gold nanorod. A transient in-plane rotation may be an indication that the nanorod is tethered to the cell membrane by multiple binding sites. However, this kind of multi-point binding is highly dynamic and can dissociate at the next few milliseconds as indicated by the disappearance of the in-plane rotation mode.

Pearson’s correlation coefficient analysis was applied to evaluate the rotational patterns. We have shown previously that for gold nanorod’s in-plane rotation, the DIC bright and dark intensities are highly correlated with a correlation coefficient close to 1, while for the out-of-plane rotation, the DIC bright and dark intensities are anti-correlated with a correlation coefficient of −1^23^. Supplementary Fig. 10 shows the correlation coefficients of the DIC bright and dark intensities of 150 transferrin-coated gold nanorods at different stages of interacting with the cell membrane. For the nanorods having active lateral diffusion in a relatively large area at the early stage of binding on the cell membrane, the distribution of correlation coefficient is centered at 0.48 with higher proportion of smaller correlation coefficients. This indicates the nanorods have higher tendency of out-of-plane motions due to the electrostatic repulsion between the negatively charged transferrin-coated gold nanorods and the negatively charged cell membrane. At the stage when gold nanorods lose their translational freedoms, the distribution of correlation coefficient is centered at 0.65. With specific binding to cell membrane receptors, gold nanorods perform predominantly in-plane rotation. The difference in the distributions of the correlation coefficients at different binding stages is statistically significant with a paired Student’s *t*-test (*p* < 0.001).

### Characteristic rotational motions during endocytosis

For all the nanorods that were eventually internalized, their fast rotational motions slowed down drastically at a certain point of time, an indication that the nanorods were enclosed in clathrin-coated pits (CCPs). The nanorods then all experienced a relatively quiet period of several tens of seconds, during which the nanorods showed no active rotational diffusion but rather slow, directed orientation changes, mostly likely under the influence of endocytic protein machinery. At the end of the quiet period, the nanorods restored active rotational diffusion, indicating the nanorod-containing vesicles were detached from the membrane.

This active rotation—quiet period—active rotation pattern was observed for all of the recorded (>100) endocytosis cases. In the case shown in Fig. 3, the co-localization of the fluorescent CCP and the nanorod was observed at 530.8 s (Fig. 3e). Then the nanorod stopped active rotational diffusion at ~536.5 s, followed by a 20-s quiet period (Fig. 3f and Supplementary Movie 5). During the quiet period, the nanorod displayed a nearly constant bright intensity but slow changes in the dark intensity from 542 to 553.7 s. At 553.7 s, the DIC image of the nanorod changed from completely dark to completely bright within only 2–3 frames (less than 100 ms), indicating the nanorod suddenly restored active rotational diffusion. The translational motion of the vesicle followed at 559.9 s, showing a slow moving away from the original entry spot. At 570.2 s (~16.5 s after the detachment event), we observed that the clathrin coat already disassembled from the vesicle (Supplementary Movie 6). This nanorod-containing vesicle was continuously followed until it was actively transported inside the cell at ~599.4 s (Supplementary Movie 7).

### Intracellular transport in 3D cellular environments

Following the endocytosis, the endocytic vesicles can be transported intracellularly by motor proteins on the microtubules and actin filaments. Within the first ~45 min following the cellular uptake, the endocytosed substances mostly reside in early endosomes that are about 50–100 nm in diameter^47, 48^. As discussed extensively in our previous publication^22^, the transferrin-coated gold nanorods are bound to the membrane receptors and tightly wrapped by the vesicle membranes. These nanorods generally lose the independent rotational freedoms relative to the vesicles; therefore, they can be excellent reporters of translational and rotational motions of early endosomes being transported by motor proteins.

In the present study, the 5D-SPT technique enabled us to reveal the full motions of nanorod-containing vesicles in living A549 cells with unprecedented accuracy. It should be noted that in order to track the movement in the *z*-axis, the automatic feedback control was always activated when studying intracellular transport. This was different from the intermittent activation when studying endocytosis, during which the nanorods were in or on the membrane with little vertical movement other than focus drift.

**Fig. 4:**
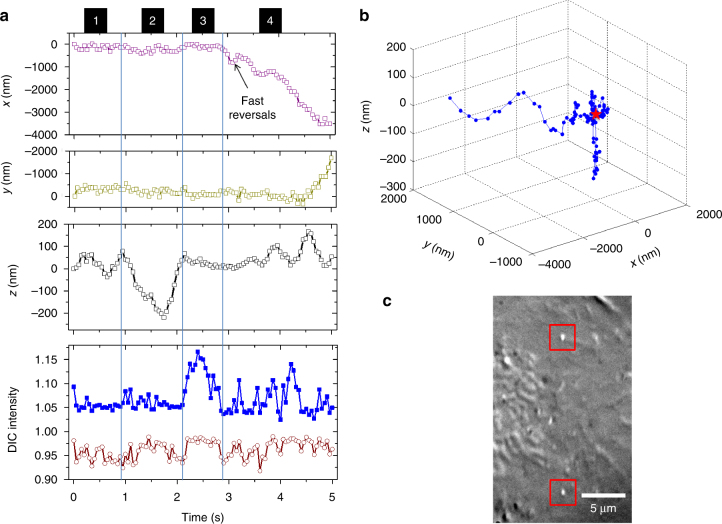
Intracellular transport of a nanorod-containing vesicle in a living A549 cell. **a** The time series of the *x*, *y*, and *z* displacement, relative DIC intensities and orientation angles of the gold nanorod. The entire recording is divided into four segments as labeled: (1) searching for a track; (2) vertical transport; (3) a big turn; (4) lateral transport with twisted up-and-down motions. **b** The 3D trajectory of the cargo. The starting position (0, 0, 0) is highlighted by the red star. **c** Parallax-DIC image of the 5D-SPT of the gold nanorod of interest as indicated by the red boxes

One representative example is shown in Fig. 4 and Supplementary Movie 8. The random but confined rotational motions of the cargo within the first second of the recording likely reflect the process of the cargo searching for a cytoskeleton track. Starting at ~1.0 s, the cargo moved nearly straight along the *z*-axis for ~300 nm and returned at 2.1 s, suggesting a possible back-and-forth movement along a track that is perpendicular to the image plane. Then the cargo made a big turn during a pause lasting from 2.1 to 2.9 s, followed by lateral movement of over 5 µm in the last 2 s of the recording. The big turn was likely due to the cargo switching from the perpendicular track to a horizontal one. The cargo also underwent a fast reversal (going back and forth) at 3.0 s, while the rotational behaviors of the nanorod could be observed, possibly because of the rearrangement of motors during which the tension on the cargo was weakened^22^. These observations combine to render the process of cargo transport inside a living cell with great details: cargo searching for a track, directional transport, track-switching, and pause. Three additional examples are provided in Supplementary Figs. 11–13 and Supplementary Movies 9–12.

An interesting sequence of movement was observed at 3.6–5.0 s in Fig. 4, when the cargo changed its moving direction laterally and vertically, accompanied by changes in its orientation. The sinusoidal-shaped vertical displacements were in the range of 100–160 nm, in good agreement with the span of the 80 nm long gold nanorod when it tumbled around a microtubule track of ~25 nm in diameter. Combined with the nearly linear lateral (*x* and *y*) trajectories, a likely explanation is that the cargo moves along the microtubule track with a twister-like motion. This phenomenon may be ascribed to microtubule bending (a curved conformation of the tubulin dimers)^49, 50^. Although the steric hindrance induced by the crowded cellular environment is another plausible explanation, the smoothness of the 3D trajectory suggests a low probability of a completely random maneuver. This type of motion in live cells is not attainable with other SPT methods.

## Discussion

The findings in the current study disclose characteristic rotational motions of nanorod cargos at different endocytic stages: active rotation, quiet period, and active rotation again with diffusion and transportation. Combining with the dynamic clathrin aggregation observed in fluorescence mode, this rotation-based model not only supports the current endocytosis models derived from the spatial and temporal organization of modular protein molecule groups^51–57^, but also provides new insights in the understanding of endocytosis progression. In the early phase of the modular model, the coating module—proteins involved in cargo recruitment and coat formation—forms on the membrane. This can be confirmed in our fluorescence observations that clathrin assembles at this early stage. Further, the 5D-SPT technique reveals that in this phase, the loss of translational freedom of the cargo does not coincide with the loss of rotational freedom. The nanorod kept rotating after apparent clathrin aggregation proves that the complete fixation of the cargo on the cell membrane happens after the initiation of the endocytosis process. The rotational motion eventually comes to a complete stop, which may be attributed to three factors: (1) the clathrin coat wrapping around the CCP to greatly reduce the fluidity of lipid membranes; (2) the polymerization of actin filaments to anchor the CCP; and (3) the polymerization of dynamin at the neck of the CCP to fully encapsulate the nanorod inside the CCP. Further confirmation of the roles of actin and dynamin is currently underway. The 5D-SPT technique provides a unique opportunity to study the functions of different assisting proteins and how different coatings facilitate or inhibit endocytosis.

On the other hand, we demonstrated the direct visualization of the full motions of the cargo in the 3D cellular environment during the intracellular transport. Future efforts along this line may provide new evidence to complete our understanding on the competition and coordination of kinesin and dynein motor proteins in live cells. Furthermore, a unique trait of the 5D-SPT technique is that the recorded moving traces reflect the 3D organization of the cytoskeleton tracks inside the cell. Although the current set-up does not allow the visualization of the 3D structures of the cytoskeleton, it is promising that the 5D tracking of intracellular transport can be combined with super-resolution imaging of cytoskeleton structures^58^ so that the transport mechanism of cargos can be better elucidated. Critical future steps are to combine the 5D-SPT of plasmonic nanoparticle probes with the fluorescence imaging of associated proteins, such as tau^59–61^ and dynactin^62^, to understand the regulated intracellular transport at the molecular level.

In summary, the parallax-DIC microscope and accompanying tracking program have been developed to realize automatic 5D-SPT in live cells. The axial movement of gold nanorod probe is detected by monitoring the distance between two half-plane images generated with a wedge prism. A vertical localization precision of 17 nm has been achieved. As demonstrated through the live cell SPT experiments, the 3D trajectories and DIC intensities incorporating rotational information of gold nanorod-containing vesicles can be correlated to elucidate different stages of the CME and the intracellular transport events in unprecedented detail, which makes the 5D-SPT technique a promising tool to shed new light on the working mechanisms of molecular motors in live cells.

## Methods

### Imaging system

The Nikon Eclipse 80i upright microscope used in the present study supports DIC mode and epifluorescence mode. The new parallax-DIC mode was enabled by adding a custom-cut wedge prism (0.5° wedge angle deviation, Edmund, Barrington, NJ) (Fig. 2) in the light path.

When the microscope was operated in epifluorescence mode, the sample was excited by a mercury lamp. When the microscope was operated in DIC mode, a set of Nomarski DIC optics (including two Nomarski prisms, two polarizers, and a quarter-wave plate) were used. A halogen lamp was used to illuminate the samples through an oil immersion condenser (numerical aperture (NA) 1.40) and the optical signals were collected with a 100× Plan Apo VC NA 1.40 oil immersion objective. The DIC images at a selected wavelength were collected by inserting the corresponding bandpass filter (Semrock, Rochester, NY) into the light path in the microscope.

In parallax-DIC mode, the wedge prism set was placed at the back focal plane of the objective in the DIC configuration. The addition of the wedge prism split the light in half, resulting in the formation of two half-plane images. Details of the implementation and evaluation of the 5D-SPT technique are presented in the Supplementary Note 1.

A Photometrics Evolve 512 EMCCD camera was used for image and video recordings. A rotary motor from Sigma Koki (model no. SGSP-60YAM) was coupled to the microscope stage to control the *z*-position of the sample. A heating stage was used when necessary. The 5D-SPT technique was implanted by compiling the correlation mapping and *z* calibration into a NIH ImageJ/µManager^63, 64^ plugin using Java. MATLAB and NIH ImageJ were used to analyze the collected images and videos.

### Preparation of transferrin-coated gold naonrods

Gold nanorods used in the experiments were all obtained from Nanopartz (Salt Lake City, UT). The purchased gold nanorods were stabilized in cetyltrimethylammonium bromide solution. Gold nanorods with aspect ratio range from 1.36 to 3 were tested and the optimal sizes were used for the experiments as specified. To functionalize the surface of gold nanorods with transferrin (CAS: 11096-37-0, Sigma-Aldrich), a *N*-hydroxylsuccinimide polyethylene glycol thiol (NHS-PEG-thiol) linker (CAS: 947601-98-1, Sigma-Aldrich) was used by following a published protocol^65^. The NHS-PEG-thiol linker has both disulfide and succinimidyl functionalities for chemisorption onto gold and covalent coupling of transferrin and/or other proteins. As for the gold nanorods, excessive surfactant was first removed from 1.0 mL gold nanorod solution by centrifugation at 2000×*g* for 10 min and resuspended in 18.2 MΩ Milli-Q water. The freshly prepared gold nanorod solution was then mixed with 10 µL 20-mM NHS-PEG-thiol solution (in dimethyl sulfoxide) and reacted for 1.5 h. After that, the solution was centrifuged at 2000×*g* for 10 min and resuspended in Milli-Q water to remove excess NHS-PEG-thiol. An aliquot of 20 µL of 2 mg ml^−1^ transferrin in phosphate buffer saline (PBS, pH 7.4) was added to the gold nanorod solution and reacted for 3 h. The transferrin-coated gold nanorods were then centrifuged at 2000×*g* for 10 min and resuspended in Milli-Q water before use.

### Cell culture for live cell imaging

A549 human lung cancer cells (CCL-185, ATCC) were plated in a T25 cell culture flask (Corning, NY) and grown in complete cell culture medium containing F-12K medium (30-2004, ATCC) and 10% fetal bovine serum (FBS, 30-2020, ATCC) in a cell culture incubator (37 °C, 5% CO_2_). An appropriate amount of cell suspension solution was subcultured onto a 22 × 22 mm poly-L-lysine (PLL)-coated coverslip, housed in a 35-mm Petri dish (Corning, NY) and kept in the cell culture incubator for 1 h to allow the cells to attach to the coverslip. After that, 1.5 mL complete cell culture medium was added to immerse the coverslip. The cells were ready for use when the cell culture reached about 70% confluency. Two pieces of double-sided tape served as spacers were attached on top of a pre-cleaned glass slide. A coverslip with cells was placed on top of the tapes with two edges to form a chamber, with the cell side facing the glass slide. The two open sides of the chamber were sealed by nail polish to prevent evaporation. The sandwiched chamber was made right before imaging.

### Transfection of A549 cell to express clathrin-LCa-EYFP

*E. Coli* bacteria with clathrin-LCa-EYFP plasmid was acquired from Addgene (Plasmid 21741, Cambridge, MA)^41^. The plasmid was extracted from the bacteria by using plasmid extraction kit (cat. 27104) and the corresponding protocol from QIAGEN (Valencia, CA). The A549 cells were pre-grown on coverslip in a Petri dish for 24 h in the incubator. The EYFP-clathrin plasmid was mixed with the transfection reagent (FuGene6, Roche Applied Science, Indianapolis, IN) in serum-free cell culture media first and introduced into the Petri dish containing the cells. After additional 24–48 h incubation, the cells were ready to use. In control experiments, the cells were incubated with hypertonic media (0.45 M Sucrose) or clathrin coat disruption drug (10 µg ml^−1^ Chlorpromazine Chloride) at 37 °C before exposure to the surface-modified gold nanorods.

### siRNA knockdown

The siRNA oligonucleotide sequence 5′-GAGCAUGUGCACGCUGGCCA-3′ targeting μ2-2 and the control siRNA oligonucleotide sequence 5′-AACACAGCAACCUCUACUUGG-3′ were both purchased from Dharmacon, Inc. through custom synthesis option. The siRNA knockdown was performed by using oligofectamine (Invitrogen) following the manufacturer’s instructions. Briefly, A549 cells were plated onto 35-mm Petri dishes 1 day before transfection in growth medium without antibiotics so that cells would be 30–50% confluent at the time of transfection. For each dish, 3 μL oligofectamine was diluted and mixed gently by pipetting up and down in Opti-MEM^®^ I Medium (Life Technologies) to a final volume 15 μL and incubated for 10 min at room temperature, while 5 µL of a 20 µM stock oligonucleotide (10 nmol synthetic siRNA in 500 µL of 1× siRNA buffer) was diluted and mixed gently in Opti-MEM^®^ I Medium to a total volume of 185 µL. Then the two solutions were mixed gently and incubated at room temperature for 30 min. This mixture was then combined with 800 µL of Opti-MEM^®^ I Medium and added to the cells after washing once with Opti-MEM^®^ I Medium. The final concentration of siRNA was 100 nM. An aliquot of 500 µL of growth medium without antibiotics was added after incubating the cells with the transfection mixture in the cell incubator for 4 h. The cells were kept in the incubator until they were trypsinized the next day. Second transfection was then performed following the same protocol as described above 1 day after replating. Cells were trypsinized 48 h after the second transfection, replated on 22 × 22 mm coverslips and allowed to grow one additional day for DIC microscopy experiments.

Disruption of AP-2-dependant endocytosis of transferrin-coated gold nanorods after siRNA treatment were assessed by DIC microscopy. Cells were transfected and treated as described above. An aliquot of 25 µL of 0.05 nM transferrin-coated gold nanorod (40 × 80 nm) solution was mixed into 1 mL serum-free medium and added to the cells after washing cells with 1 × PBS and incubated at 37 °C in the cell incubator for 30 min. The cells were then washed three cycles with cold 1× PBS and fixed for 10 min at room temperature in freshly prepared formaldehyde solution containing 3% paraformaldehyde and 0.1% glutaraldehyde, followed by three cycles of 10-min washing with 1× PBS on a shaker. The uptake of gold nanorod was visualized under a DIC microscope at different wavelengths and z-stacks were acquired using the objective scanner and reconstructed in Amira, a 3D visualization and analysis program.

### Data availability

All relevant data are available from the authors on request.

## Electronic supplementary material

Supplementary Information

Description of Additional Supplementary Files

Supplementary Movie 1

Supplementary Movie 2

Supplementary Movie 3

Supplementary Movie 4

Supplementary Movie 5

Supplementary Movie 6

Supplementary Movie 7

Supplementary Movie 8

Supplementary Movie 9

Supplementary Movie 10

Supplementary Movie 11

Supplementary Movie 12
