# Supplementary material for: Characteristic rotational behaviors of rod-shaped cargo revealed by automated five-dimensional single particle tracking
Source: Nat Commun. 2017 Oct 12;8:887. doi: 10.1038/s41467-017-01001-9 (PMC5638882; doi:10.1038/s41467-017-01001-9)
Supplement: Supplementary file 1 — Supplementary Information [file 41467_2017_1001_MOESM1_ESM.pdf]

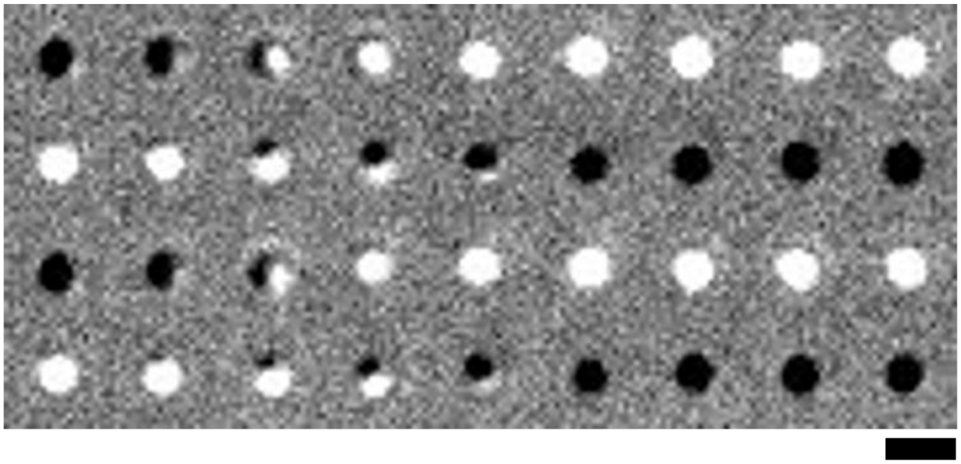

Supplementary Fig. 1. DIC images of a gold nanorod at different orientations. DIC images of a 40 nm × 80 nm gold nanorod illuminated at 660 nm (longitudinal LSPR mode) at different orientations with exposure of 33 ms exhibit periodic image pattern changes. Scale bar is 1 µm. The nanorod was placed on a rotating stage and imaged at an interval of 10°. The normalized bright intensity I_{Bright} is defined as:

$$I_{Bright} = \frac{I_{Max} - I_{Bkgd}}{I_{Bkgd}},$$

and the normalized dark intensity I_{Dark} is defined as:

$$I_{Dark} = \frac{I_{Bkgd} - I_{Min}}{I_{Bkgd}},$$

where I_{Bkgd} is the background intensity level near the particle; I_{Max} and I_{Min} are the maximum and minimum intensities of the particle, respectively.

The bright and dark intensities of a gold nanorod in each frame of a recorded DIC image stacks can be obtained simultaneously. The orientation of the gold nanorod can be determined by solving the simplified equations:[1-3]

$$I_{\mathrm{bright}} \approx 1 + \cos^2\psi \sin^4\phi$$
$$I_{\mathrm{dark}} \approx 1 - \cos^2\psi \cos^4\phi$$

where ϕ is the azimuthal angle and ψ is the elevation angle as defined in **Fig. 1A**.

Thus, the normalized DIC bright and dark intensity trace contains orientation information of the gold nanorod at each frame which reflects the rotational dynamics of the gold nanorod.

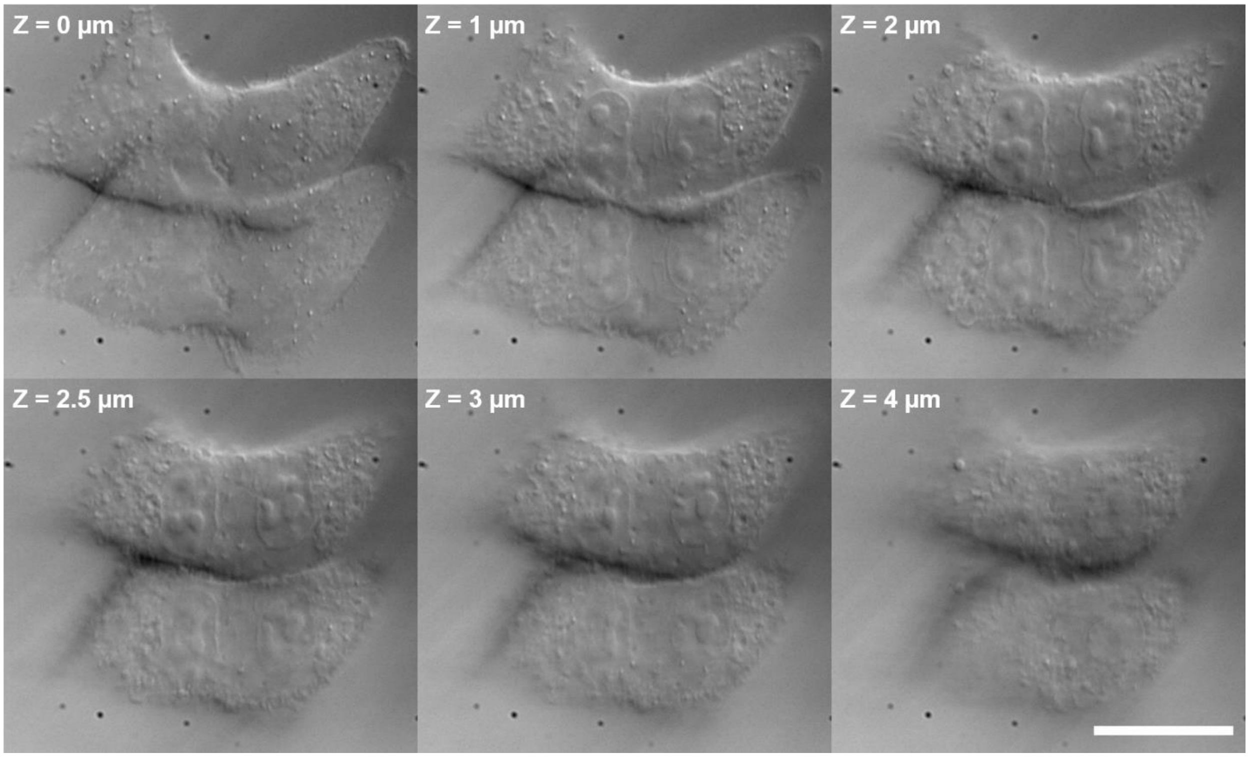

Supplementary Fig. 2. Parallax-DIC images of A549 cells at different vertical positions. The cell was scanned from bottom (the coverslip side) to top at 20 frames per second. The vertical position $z = 0$ is set arbitrarily. The scale bar is 20 μm. The overlapped region of the two half-plane images should be avoided in picking target objects to ensure high reliability and sensitivity in the 5D-SPT experiments.

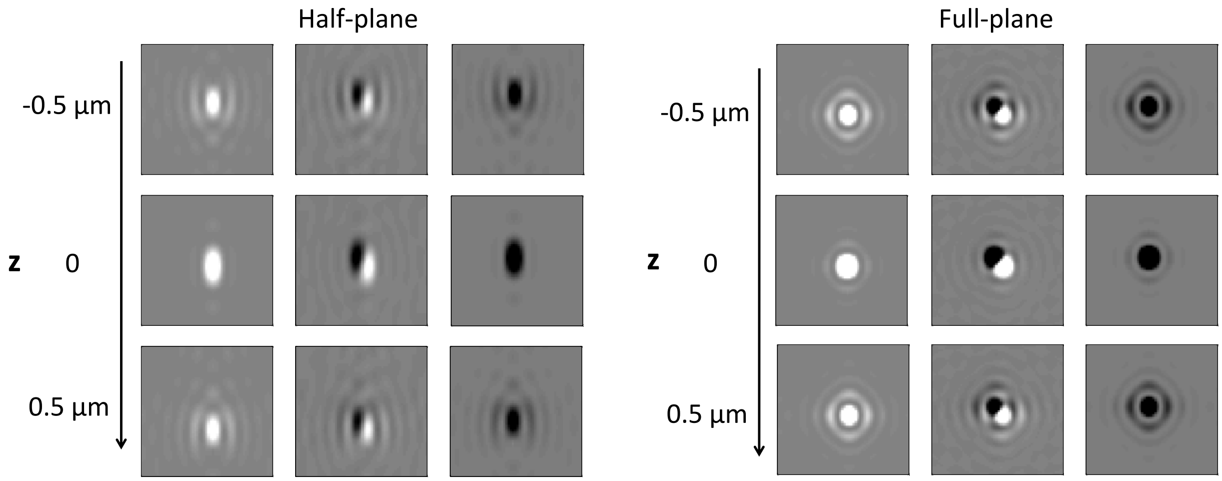

Supplementary Fig. 3. Computer simulated half-plane and full-plane images of gold nanorod showing bright (left), half-bright-half-dark (middle) and dark (right) images at different *z* positions.

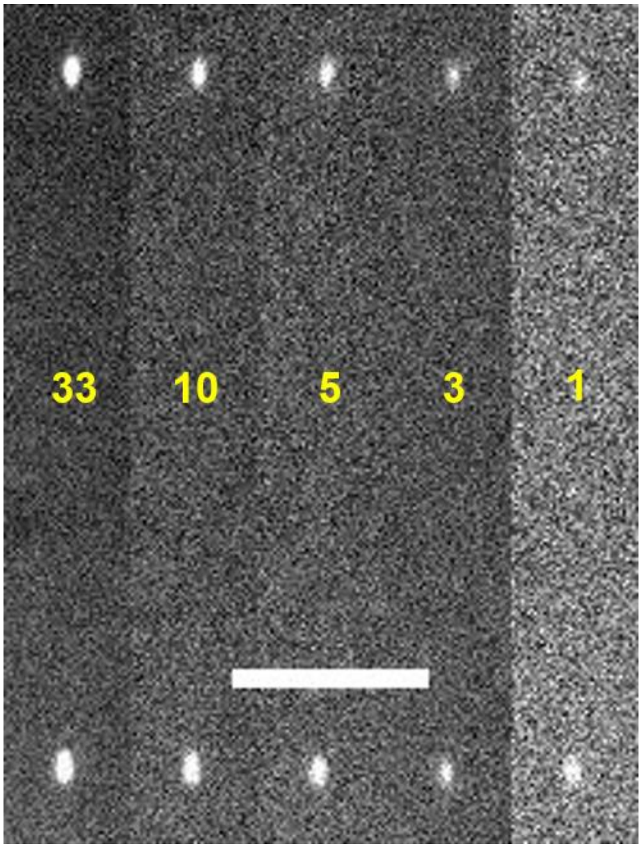

Supplementary Fig. 4. Parallax-DIC images at different exposure times. A series of Parallax-DIC images of a 40 nm×80 nm gold nanorod taken at different exposure times (33 ms, 10 ms, 5 ms, 3 ms, and 1 ms). Scale bar is 5 µm. The exposure time of 33 ms was routinely used for video rate recording while assuring good signal to noise ratio for accurate orientation determinations in 5D tracking.

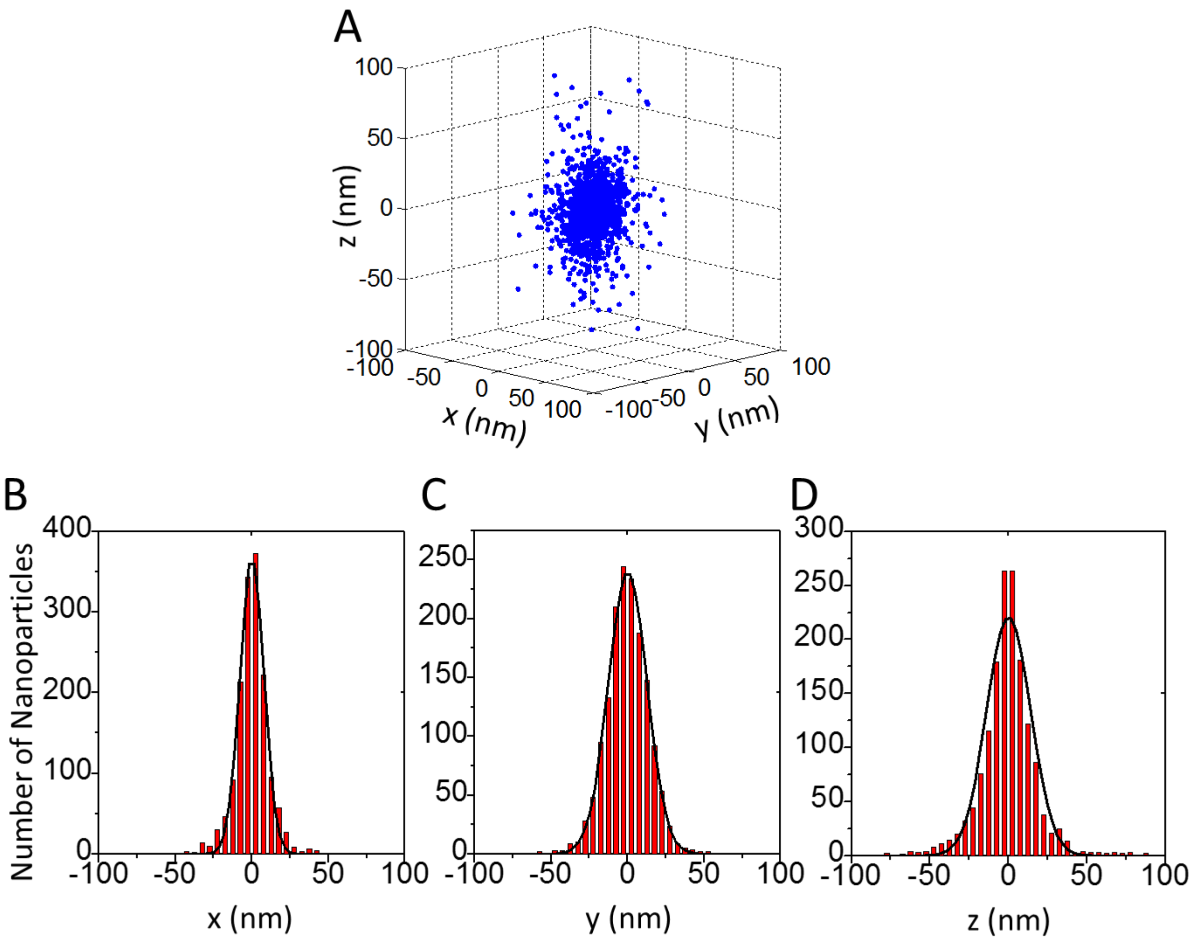

Supplementary Fig. 5. The precision measurements of the 3D localization. (A) 3D localization distribution of the gold nanorod at the temporal resolution of 30 ms. (This panel is identical to **Fig. 1D** in the main text. It is shown again here for convenience to see the distributions in the *x*, *y*, *z*-axes.) **(B-D)** Histograms of the localization distribution along *x* **(B)**, *y* **(C)** and *z* **(D)** directions are fitted with Gaussian functions that yield standard deviations of 11 nm in *x*, 14 nm in *y*, and 17 nm in *z*.

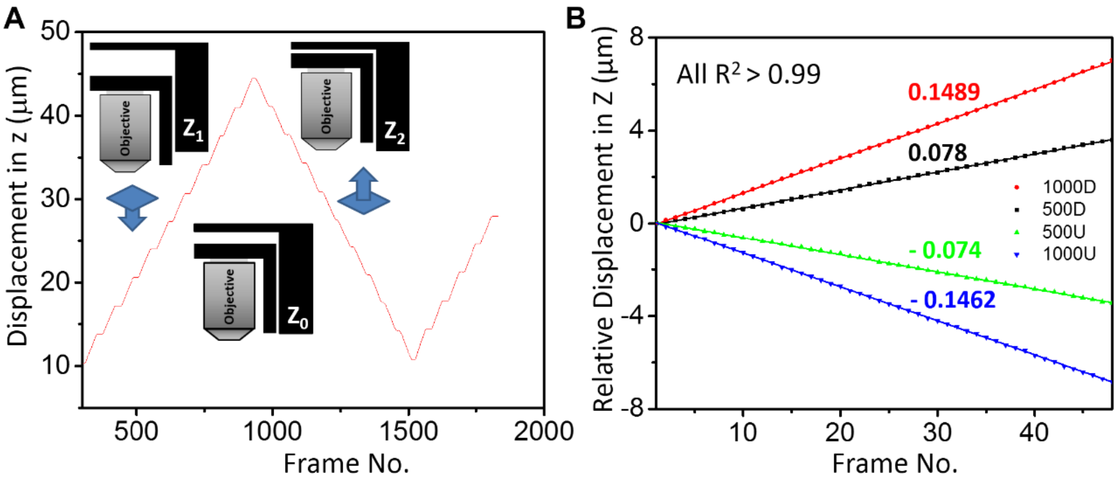

Supplementary Fig. 6. Evaluation of the performance of the 5D-SPT technique. (A) Response of the objective scanner to the sample stage movement in both directions in multiple cycles (10 s per cycle) at 0.35 μm/s. **(B)** The ability of autofocusing of 5D-SPT technique. Solid red circles (1000D) and solid black squares (500D) corresponding to the z axial of objective scanner increase when microscope sample stage was moved down at 1000 and 500 steps per second (0.70 μm/s and 0.35 μm/s) while solid green ascending triangle (500U) and solid blue descending triangle (1000U) refer to z axial of objective scanner decrease when sample stage was moved up at 500 and 1000 steps per second. These data points were all well fitted with linear lines and the coefficient of determination (R^2) were all greater than 0.99. The slopes were labelled with same color in the figure.

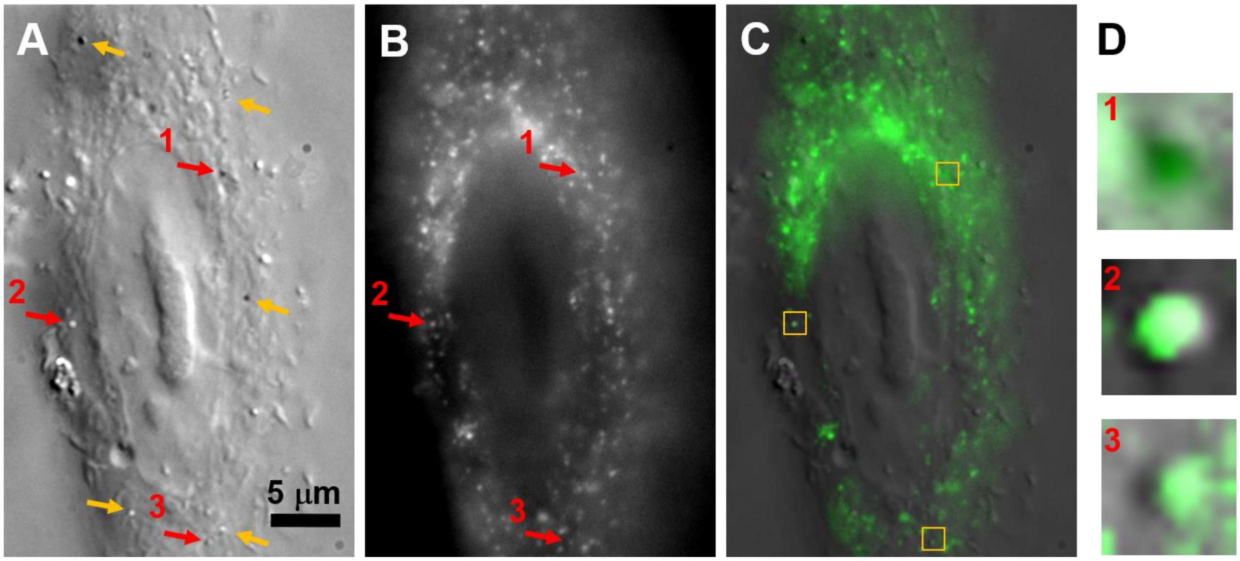

Supplementary Fig. 7. Co-localization of the gold nanorods and EYFP-clathrin-coated pits on a transfected A549 cell membrane. **(A)** DIC image, **(B)** Fluorescence image, **(C)** Overlapped DIC and fluorescence image, and **(D)** Expanded images of the framed areas in **(C)**. The three nanorods co-localized with fluorescent clathrin coats were labeled with red arrows and the other five nanorods without co-localized fluorescence were labeled with orange arrows. The integration time was 300 ms for the fluorescence image and 30 ms for the DIC image.

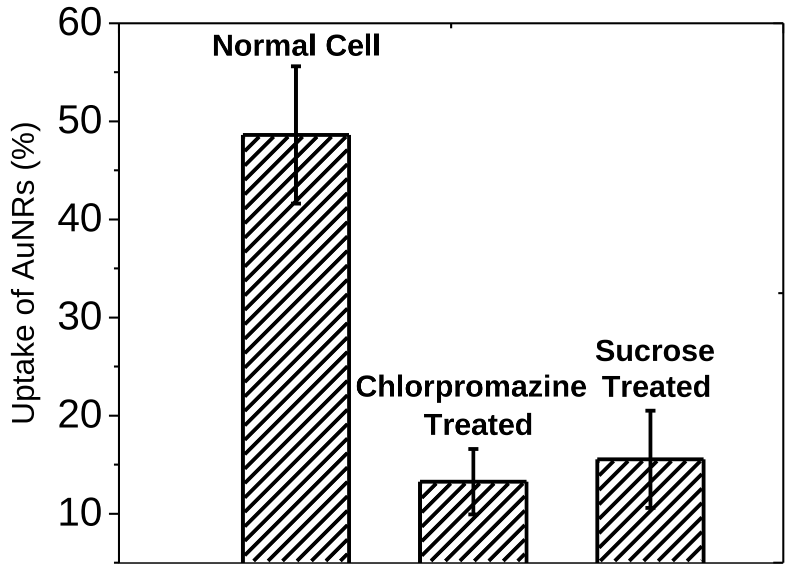

Supplementary Fig. 8. Gold nanorod uptake comparison under different drug treatments. Comparison of gold nanorod uptake within 2 hours of observation among normal cells, Chlopromazine treated cells and Sucrose treated cells. Significant decreases of gold nanorod uptake was found in Chlopromazine treated cells (13%) and Sucrose treated cells (15%) compare to normal cells (49%). (Error bar ± standard deviation, n=35)

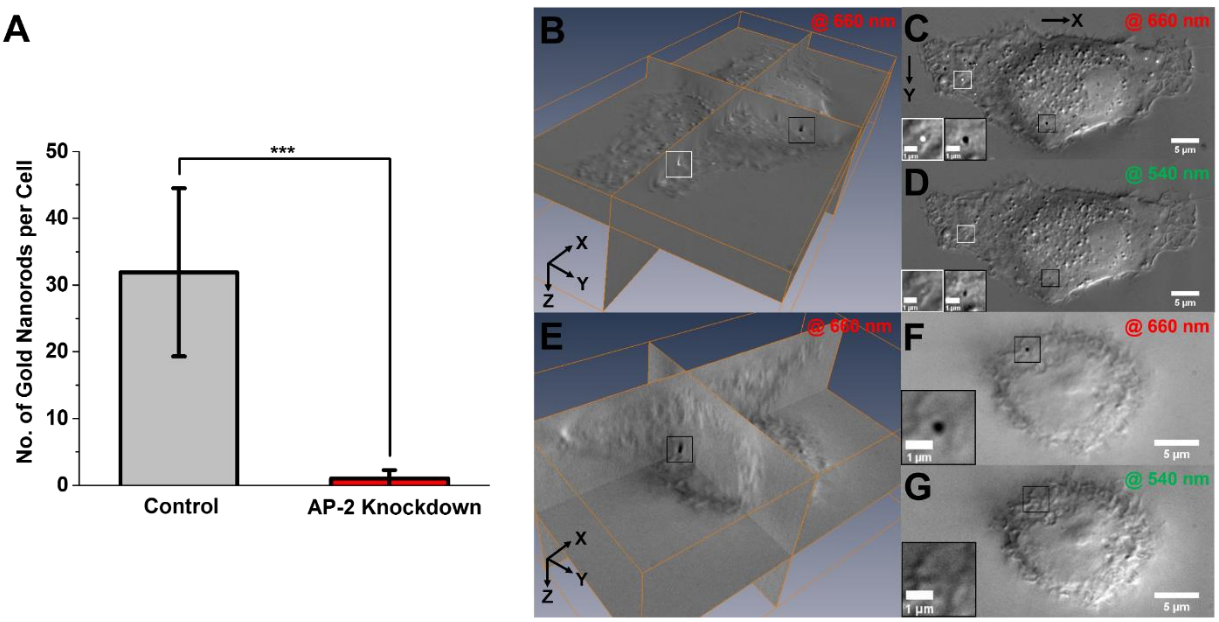

Supplementary Fig. 9. Evaluation the effect of siRNA treatment on the AP-2-dependant cellular uptake of transferrin-coated gold nanorods. (A) Comparison of the numbers of the transferrin-coated gold nanorods uptake per cell in control cells (gray) and AP-2 μ2 targeting siRNA treated cells (red). The average of 31.9±12.6 transferrin-coated gold nanorods were observed in control cells and only average of 1.1±1.2 nanorod was found in siRNA treated cells. (Error bar ± standard deviation, n=20) At least 20 randomly imaged cells were used in each group. Student's t test was used to evaluate the data. p value < 0.001 is indicated by ***. 3D reconstructions of DIC z-stack images of one of the representative examples of **(B)** control cell and **(E)** siRNA treated cell in Amira. The wavelengths used were indicated in each panel. The z-stacks were acquired by scanning through the whole cell using the high precision objective scanner. The top of the boundary box in **(B)** and **(E)** were close to the coverslip surface where the cells adhere to. **(C)**, **(D)** and **(E)**, **(F)** are the DIC images corresponding to the XY plane in **(B)** and **(E)**. 16 transferrin-coated gold nanorods were observed in one focal plane around 3 μm inside of the control cell (the approximate distance from the coverslip surface to the XY plane of the image in **(B)**) under the LSPR wavelength of 660 nm in **(C)** and the zoomed in DIC images

of the transferrin-coated gold nanorods are presented in the inserts corresponding to highlighted white and black boxes in cell with the same colors. When the same regions were examined at 540 nm in **(D)**, the signal (contrast) is rather weak. Only 1 transferrin-coated gold nanorod was found around 9 μm inside of the siRNA treated cell and it was differentiated by imaging under different wavelengths in **(F)** and **(G)**. In fact, this is the only nanorod that was found in the whole cell.

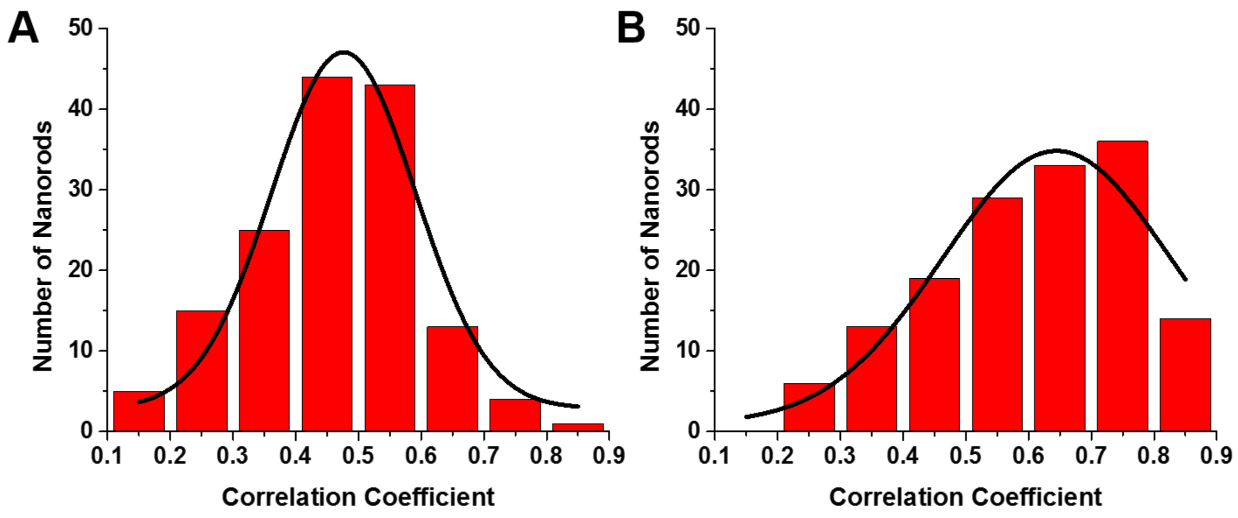

Supplementary Fig. 10. Histogram of the correlation coefficients calculated from 200-frame movies for 150 transferrin-coated gold nanorods. (A) when gold nanorods perform active lateral diffusion in a relatively large area at the early stage of binding on the cell membrane and **(B)** when these nanorods gradually lost their translational freedoms but still rotating in a relatively confined area on the cell membrane. The distributions were centered at **(A)** 0.48 and **(B)** 0.64, respectively. The perfect in-plane rotation yields a correlation coefficient value of 0.88 for DIC bright and dark intensity. The difference in the distributions of the correlation coefficients at different binding stages is statistically significant calculated with a paired Student's t-test ($p<0.001$).

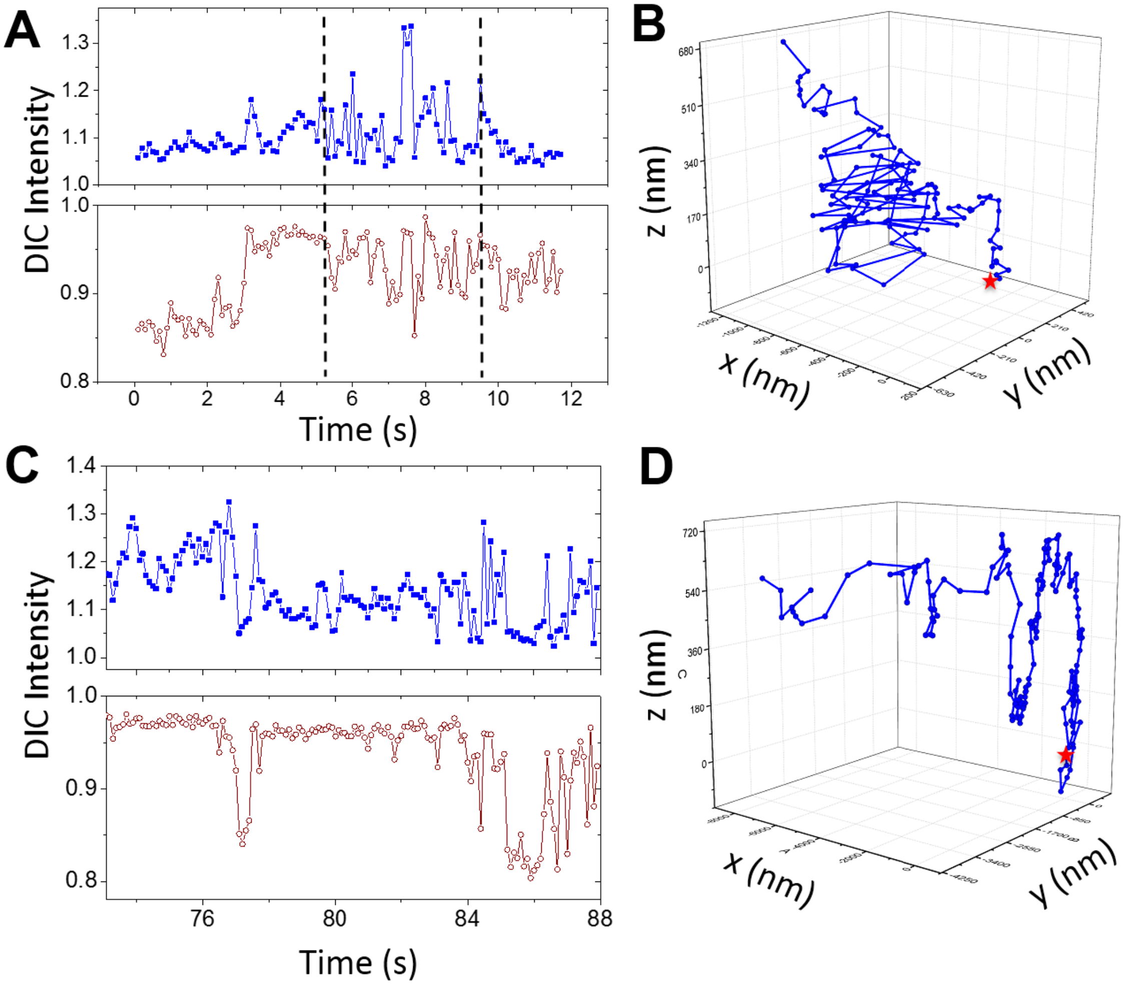

Supplementary Fig. 11. **A complete clathrin-mediated endocytosis event of a gold nanorod using 5D-SPT technique.** The gold nanorod was monitored from the early stage of binding to the cell membrane **(A)** and **(B)** until being endocytosed and then transported over a long distance **(C)** and **(D)**. Corresponding movies are presented in **Supplementary Movies 9** and **10**. Time 0 was arbitrarily set to the beginning of 5D-SPT which was shortly after the gold nanorod appeared on the cell surface.

(A) DIC bright and dark intensities of the gold nanorod at the early stages of binding (**Supplementary Movie 9**). **(B)** Corresponding 3D trajectory of the tracking in **(A)**. The gold

nanorod performed mostly out-plane rotation from the beginning of the video to 5.1 s, indicating that gold nanorod had very weak interaction with cell membrane. From ~5.1 to 9.5 s, the Parallax-DIC bright and dark intensity traces showed correlated changes. This suggested the likely clathrin aggregation around transferrin-coated gold nanorod, which induced the binding between gold nanorod and cell membrane. However, the rotational freedom of gold nanorod was still permitted. **(C)** DIC bright and dark intensities of the gold nanorod went through endocytosis and intracellular transport (**Supplementary Movie 10**). **(D)** Corresponding 3D trajectory of the tracking in **(C)**. The maturation of CCP and the subsequent endocytic process eventually restrained the in-plane rotation of the gold nanorod. A big angle change was observed from ~76.7 to 77.6 s as can be seen from the parallax-DIC image pattern changes. Then, the gold nanorod went through a quiet period until ~83.9 s. CME was proposed to happen during 76.7 to 83.9 s. In this time period, the cargo was assembled with motor proteins and the active intracellular transport occurred from 83.9 to 85.4 s, the gold nanorod was transported over 1.8 µm in a speed of ~1.2 µm/s. Interestingly, the gold nanorod stop for about 0.8 s at one location and then changed direction slightly and actively transported for another ~5.9 µm in a speed of ~3.5 µm/s. A closer scrutiny of the recorded Parallax-DIC movie suggested that the gold nanorod was first transported into a relatively crowed cellular environment and resulted in the pause of the transportation. It was able to quickly get over the obstacle, and find another track to continue the transport process.

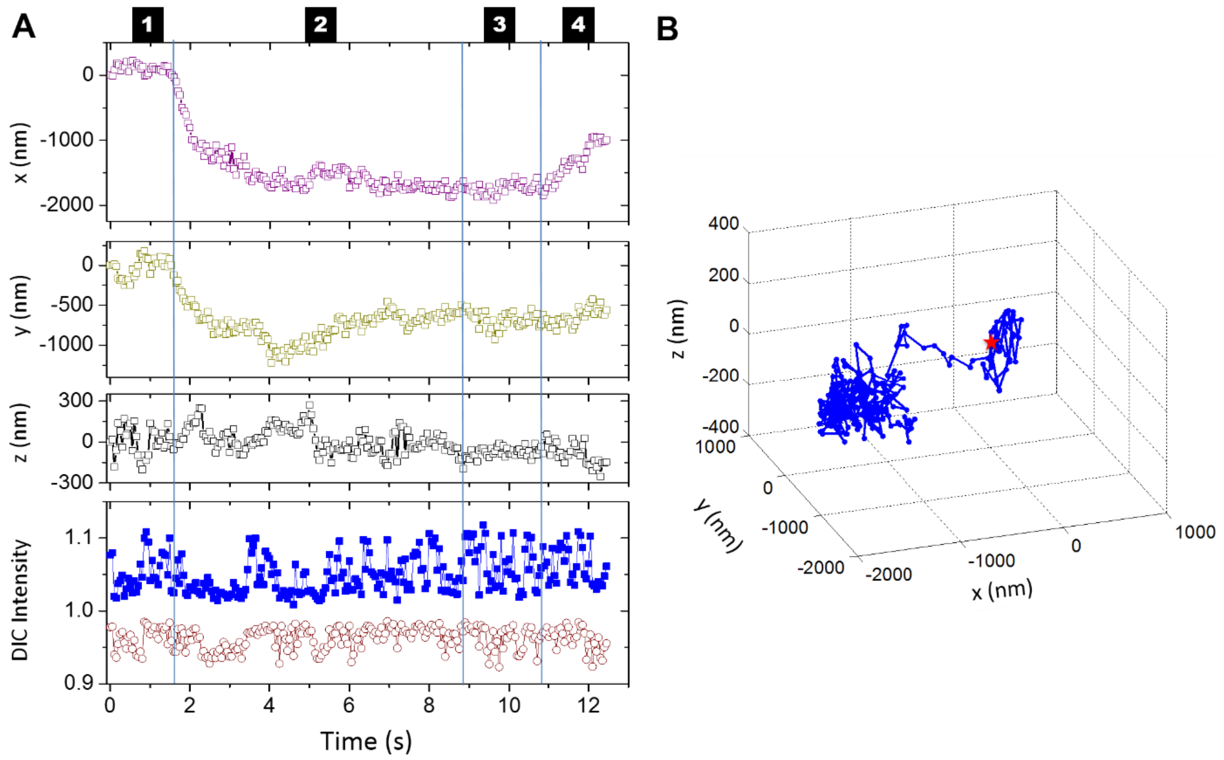

Supplementary Fig. 12. Intracellular transport of a gold nanorod-containing vesicle. (A) The *x*, *y* and *z* displacement (shown in purple, dark yellow, and black, respectively) and the relative DIC intensities of the nanorod moving inside of a live A549 cell. The bright part and dark part intensities are shown in blue and wine, respectively. The blue lines divide the movement into four segments: The particle (1) performed regional random diffusion; (2) transported along the microtubule track with twisted up-and-down motions; (3) resumed regional random diffusion; (4) underwent directional transport. **(B)** The 3D trajectory of the nanorod. The starting position (0, 0, 0) is highlighted by the red star.

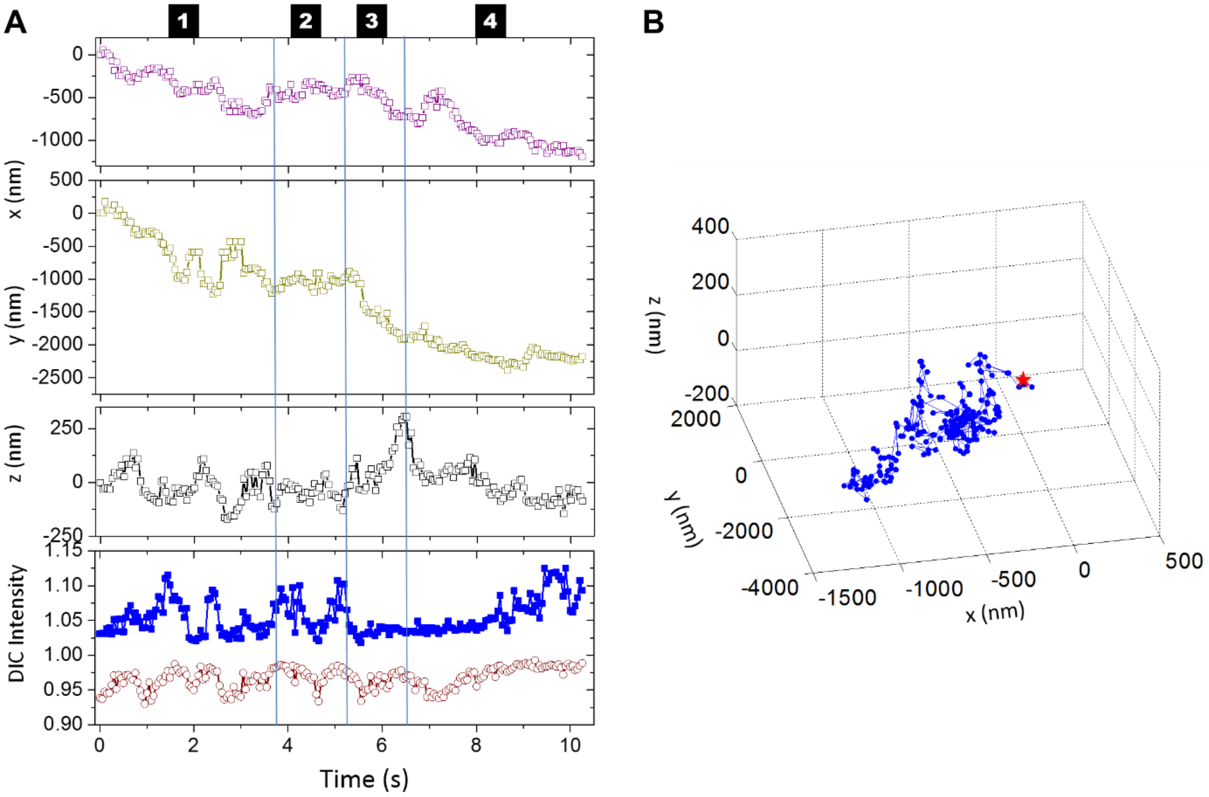

Supplementary Fig. 13. Another example of intracellular transport of a gold nanorod-containing vesicle. (A) The *x*, *y*, and *z* displacement (shown in wine, dark yellow, and black respectively) and the relative DIC intensities of the nanorod moving inside of a live A549 cell. The bright part and dark part intensities are shown in blue and red respectively. The cargo (1) transported with twisted up-and-down motions; (2) paused with rotation; (3) underwent directional transport; (4) transported with twisted up-and-down motions again. **(B)** The 3D trajectory of the nanorod. The starting position (0, 0, 0) is highlighted by the red star.

SUPPLEMENTARY NOTE 1

Correlation Mapping and Calibration of Z-localization

An objective scanner (Physik Instrumente, Model P-721.CDQ) was installed on the microscope, mounted with the 100× Plan Apo VC 1.40 oil immersion objective, and connected to a Piezo position servo controller (Physik Instrumente, Model E-709). Movies of the vertical scans of the fixed gold nanorods were taken while the camera and the objective scanner were synchronized at 200 ms per frame and per step of movement for calibration purpose. The step size of the vertical scan was 20 nm.

The *z*-position of the gold nanorods was calibrated against the distance between the two half-plane DIC images of a single gold nanorod. The distance between the two DIC images was measured using the correlation mapping method. At each *z*-position, one of the half-plane images was used as the model to map both of the two images. The model image was cropped and overlaid with the region of interest (ROI) that includes both images, and moved pixel by pixel and line by line to cover the entire ROI. At each position, a correlation score in the form of Pearson’s correlation coefficient was calculated as

$$p(\text{model},\text{sub-ROI}) = \frac{1}{m-1}\sum_{i=1}^{m}\frac{\left[I_{\text{model}}(i)-I_{\text{model}}(avg)\right]}{\sigma_{\text{model}}}\frac{\left[I_{\text{sub-ROI}}(i)-I_{\text{sub-ROI}}(avg)\right]}{\sigma_{\text{sub-ROI}}},$$

where the summation covers all the pixels in the model or the sub-ROI, m is the total number of pixels in the model, I is intensity, *avg* and σ denote the average and standard deviation of the intensity in the model or the sub-ROI, respectively. By weighing the p values above a threshold of the region that the image of the particle covers, the center of the particle can be determined as

$$X_{\text{center}} = \frac{\sum_{i=1}^{m} X_i\left(p_i - threshold\right)}{\sum_{i=1}^{m}\left(p_i - threshold\right)},$$

where X denotes either x or y in the calculation, and the threshold is applied to cut off the background noise. The center coordinates of the two nanorod images, (x_1, y_1) and (x_2, y_2) are thus determined. For the highest possible accuracy, the prism inserted into the light path of the microscope was well pre-aligned so that the two images were not shifted horizontally. The distance between the two images is:

$$d = |y_2 - y_1| .$$

The calibration curve of the distance d as a function of z is thus obtained from the vertical scans where each data point is the average value obtained from the gold nanorod placed at 36 orientations (0-180° with 5° intervals). The calibration data (**Fig. 1C**) result in a good linear fitting going through (0, 0), which refers to the vertical position of the nanorod in focus.

The axial position of a target object is determined from the distance between the two half-plane images. The axial localization is calibrated by scanning the focal plane of the objective vertically in 20 nm steps through the stationary gold nanorods and correlating the distance between the two images with the axial position of the sample. Each data point on the calibration curve (**Fig. 1C**) is the average distance between the two half-plane images as the nanorod is rotated 180° in 5° intervals. The distance between the two images falls in a linear relationship with the axial position of the gold nanorod when the vertical displacement is within the displayed range of -0.5 μm to +0.5 μm relative to the focal plane. For the current setup, the calibration curve returned a slope of +0.626 (d/Δz) and R^2 of 0.997.

Both correlation mapping and calibration of z-localization were compiled as a μManager plugin in the autofocusing tracking program.[4-5] Movies of the tracking, (x, y, z) relative spatial localization, and DIC bright and dark intensity traces were generated by the tracking program.

It should be noted that the two half-plane images are not identical. The image formed through the wedge prism is slightly longer along the direction perpendicular to the split plane than the other image formed in the original light path of the microscope. Small variances in the relative intensities between the two half-plane images could also result from placing the wedge prism at an imperfect position to split the light. Nonetheless, the subtle differences in image intensity and shape do not significantly affect the accuracy of the correlation mapping procedure, as shown in **Fig. 1C** that the distances between the two half-plane images are remarkably consistent for the entire angle range of 0-180°. Using a set of mirrors instead of a wedge prism has been demonstrated to be more flexible and precise to achieve parallax in fluorescence microscopy;[6] however, the presence of two Nomarski prisms makes the mirror-based design much more difficult to realize.

A common concern of the high-accuracy localization of a transition dipole is associated with the asymmetric emission/scattering intensity distribution when the dipole is titled relative to the horizontal plane.[7-8] As a result, the Gaussian fitted image center may not overlap with the actual geometric center of the probe. It is important to note that this type of localization error (on the order of a few tens of nanometers) is critical for the localization of stationary imaging probes where nanometer precision is often required.[9] However, for the dynamic SPT experiments where nearly everything is moving constantly, these small localization errors can be neglected without noticeably affecting the recorded 3D trajectories.

3D Localization of Gold Nanorods in 5D-SPT

The *z* coordinate is obtained by fitting the measured distance between the two images of the gold nanorod into the calibration curve. To measure the 3D localization precision of gold nanorods with random orientation, gold nanorods were dispersed in agarose gel. The gel matrix was made by dissolving agarose powder in 18.2 MΩ milli-Q water (2% w/w) and heating the mixture for 5 min in a water bath. 50 μL of the 40 nm × 80 nm gold nanorods was added to 1 mL of the gel when hot and the mixture was vortexed vigorously and spin-casted onto a pre-cleaned glass slide and covered by a glass coverslip. The sample slide was then cooled down and brought under the Parallax-DIC microscope and particles at different depths were imaged. A cluster of localization was obtained for each particle by determining the position of the particle in 10 frames of images. The overall 3D localization distribution was then generated by overlapping the center of mass of the localization clusters of 155 gold nanorods. (**Fig. 1D**) Standard deviations in the x, y, and z directions are thus obtained by fitting the distribution histogram of localization with Gaussian functions. These results are shown in **Supplementary Fig. 5 B-D**.

Implementation of 5D-SPT with Autofocusing

The objective scanner is set to "idle" before the camera starts to record the movies. The objective is adjusted by the user to bring the nanoparticle in focus. The initial coordinates of the target particle are defined by the user. Using the initial coordinates as the center, the tracking program defines a 7 × 7 pixel square. A hollow region is formed by framing a smaller square (4 × 4 pixel) inside the big square. The small frame is moved pixel by pixel inside the big square until the biggest difference between the mean intensity of the small square inside of the frame and the hollow region is found, where the small square covers the pixels of the highest contrast

constituting the half-plane image of the gold nanorod. The sizes of these squares are chosen to achieve the accurate localization within a time duration that is much shorter than the moving speed of the objective scanner.

The distance between the two half-plane images when the nanoparticle is in focus is measured as d_0. At a distance of d_0 from the first half-plane image, the estimated position of the second half-plane image is defined. The 4 × 4 pixel small square that covers the first half-plane image is then moved pixel by pixel to cover both itself and the second half-plane image of the nanorod, and two correlation maps are generated. The distance between the centroids of the two correlation maps is then compared with d_0 and the difference is converted to the vertical distance from the focal plane.

The autofocusing algorithm is realized via the objective scanner controlled by the PI Controller E709. The frame numbers and the distances the objective scanner travels at the corresponding frames are recorded and output in a text file. To reduce the error caused by the time delay of the adjustment of the objective scanner, the actual vertical position is retrieved as the distance the objective scanner moved from the last frame plus the relative vertical position of the nanorod converted from the distance between the two half-plane images of the current frame.

Evaluation of Autofocusing Performance

The performance of autofocusing was first evaluated by the autofocusing feedback capacity with respect to the stage movements. The microscope sample stage was coupled to a motorized rotation driver (Sigma Koki, CSG-602R) through the Nikon 80i microscope fine adjustment knob. With the autofocusing tracking program on, when the motorized rotation stage was initiated to move the sample stage up or down, the objective scanner adjusted its position, thus the position of the objective, accordingly, to maintain the nanoparticle of interest in focus.

Supplementary Fig. 6 shows the response of objective scanner when the stage was moved toward different directions with different speeds. An immobilized gold nanorod sample slide was prepared by adding about 5 μL of the above-mentioned 40 × 80 nm gold nanorod solution between a cleaned glass slide and a coverslip and sealed all the edges by nail polish. The immobilized gold nanorod sample was first found by manual adjustment to a relatively clear image, and then the autofocusing tracking was initiated to target a specific nanoparticle. When the sample stage was moved at 500 and 1000 steps per second (0.35 and 0.70 μm/s) toward both directions in z axis, the autofocusing feedback was fast enough to adjust the objective scanner accordingly as indicated from the perfect linear fitting in the figure. The slopes were 0.078 and 0.1489 when the sample stage was moved down with respect to objective. And the slopes were -0.074 and -0.1462 when it was moved upward. The coefficient of determination (R^2) of these fittings were all larger than 0.99. The response of the autofocusing tracking speed toward 0.70 μm/s z axial movement speed is far than enough to track most of the cellular processes. Also, the z axial tracking range is not restricted by the depth of field of the objective (several μm) but is only limited by the travel distance of the objective scanner (100 μm). This enables z tracking throughout the entire thickness of any kinds of mammalian cells.

Computer Simulation of Full-Plane and Half-Plane DIC Images

Computer simulation of the full-plane and half-plane PSFs of a gold nanorod was adapted from the simulation of the Parallax images of a point source.[6] The simulation is based on the simplified model that assumes the incident light from a point emitter produces a band-limited version of Fourier transform of a delta function in the back aperture of the objective. In this model, the amplitude and phase of the function keeps constant inside the circular sub-region,

while jump to zero outside. The wavefront phase disturbance due to the defocusing of the gold nanorod ΔZ is in the form of

$$2\pi r^2 \Delta Z / [\lambda f (f + \Delta Z)],$$

where λ is the wavelength, f is the focal length, and r is the radial distance from the optical axis. The wavefront phase disturbance is then added to the Fourier transform. The sum, which represents the phase component, is combined with a constant amplitude component to generate an approximation to the defocus-aberrated pupil function at the back aperture of the objective for the full-plane image. For the half-plane image, the transform was set to zero in the negative y half-plane. The square of the modulus of the Fourier transforms of these distributions yields the model PSF distributions of the simulated scattering image pattern.

The lateral scale for the simulated PSFs is defined by assigning the radius at the first minimum of the in-focus PSF a value of

$$\frac{r}{\lambda} = 0.61 / (\text{effective NA}).$$

The axial scale is defined by assigning the magnitude of defocus at the first axial minimum of the PSF a value of

$$\frac{\Delta Z}{\lambda} = 2 / (n \sin^2 \alpha).$$

where n is the refractive index of the medium surrounding the point object, and α is the objective lens acceptance half-angle.

The DIC images are then generated by integrating the scattering amplitude PSF into the DIC PSF[10]:

$$h(x, y) = (1 - R)\exp(-j\Delta\theta)k(x - \Delta x, y - \Delta y) - R\exp(j\Delta\theta)k(x + \Delta x, y + \Delta y),$$

where $2\Delta x$ and $2\Delta y$ are the shear introduced by the Nomarski prism along the x and y axes respectively, $2\Delta\theta$ is the bias retardation, where R determines the relative amplitude of the two wavefronts, and $k(x, y)$ is the amplitude PSF for the optics under coherent illumination. All the simulation figures were generated by using MATLAB.

SUPPLEMENTARY REFERENCES

1. Wang, G.; Sun, W.; Luo, Y.; Fang, N., Resolving rotational motions of nano-objects in engineered environments and live cells with gold nanorods and differential interference contrast microscopy. *J. Am. Chem. Soc.* **132**, 16417-16422, (2010).
2. Gu, Y.; Sun, W.; Wang, G.; Jeftinija, K.; Jeftinija, S.; Fang, N., Rotational dynamics of cargos at pauses during axonal transport. *Nat. Commun.* **3**, 1030, (2012).
3. Gu, Y.; Sun, W.; Wang, G.; Zimmermann, M. T.; Jernigan, R. L.; Fang, N., Revealing Rotational Modes of Functionalized Gold Nanorods on Live Cell Membranes. *Small* **9**, 785-792, (2013).
4. Schneider, C. A.; Rasband, W. S.; Eliceiri, K. W., NIH Image to ImageJ: 25 years of image analysis. *Nat. Methods* **9**, 671–675, (2012).
5. Edelstein, A.; Amodaj, N.; Hoover, K.; Vale, R.; Stuurman, N., Computer Control of Microscopes Using µManager. In *Current Protocols in Molecular Biology*, John Wiley & Sons, Inc., 2010; pp 92:14.20.1–14.20.17.
6. Sun, Y.; McKenna, J. D.; Murray, J. M.; Ostap, E. M.; Goldman, Y. E., Parallax: High Accuracy Three-Dimensional Single Molecule Tracking Using Split Images. *Nano Lett.* **9**, 2676-2682, (2009).
7. Enderlein, J.; Toprak, E.; Selvin, P. R., Polarization effect on position accuracy of fluorophore localization. *Opt. Express* **14**, 8111-8120, (2016).
8. Mortensen, K. I.; Churchman, L. S.; Spudich, J. A.; Flyvbjerg, H., Optimized localization analysis for single-molecule tracking and super-resolution microscopy. *Nat. Methods* **7**, 377-381, (2010).
9. Backlund, M. P.; Lew, M. D.; Backer, A. S.; Sahl, S. J.; Grover, G.; Agrawal, A.; Piestun, R.; Moerner, W. E., Simultaneous, accurate measurement of the 3D position and orientation of single molecules. *Proc. Natl. Acad. Sci. USA* **109**, 19087-19092, (2012).
10. Preza, C.; Snyder, D. L.; Conchello, J.-A., Theoretical development and experimental evaluation of imaging models for differential-interference-contrast microscopy. *J. Opt. Soc. Am. A* **16**, 2185-2199, (1999).
