# Supplementary material for: Characteristic rotational behaviors of rod-shaped cargo revealed by automated five-dimensional single particle tracking
Source: Nat Commun. 2017 Oct 12;8:887. doi: 10.1038/s41467-017-01001-9 (PMC5638882; doi:10.1038/s41467-017-01001-9)
Supplement: Supplementary file 2 — Description of Additional Supplementary Files [file 41467_2017_1001_MOESM2_ESM.pdf]

Description of Additional Supplementary Files

File Name: Supplementary Movie 1
Description: The Parallax-DIC images of a transferrin-coated gold nanorod at three orientations. From left to right: the nanorod is placed with its long axis at 0°, 45° and 90° relative to the bright optical axis. It shows parallax-DIC images of a stationary transferrin-coated gold nanorod at three orientations traveling smoothly from -0.4 μm below the focal plane to +0.4 μm above the focal plane at a speed of 100 nm per second with a single frame exposure time of 200 ms.

File Name: Supplementary Movie 2
Description: The transferrin-coated gold nanorod rotates fast while performing translational movement on the cell membrane at an early binding stage.

File Name: Supplementary Movie 3
Description: The transferrin-coated gold nanorod continues rotation but stops translational movement on the cell membrane at a late stage.

File Name: Supplementary Movie 4
Description: An EYFP-clathrin coated pit was found to co-localize with the transferrin-coated gold nanorod. The fluorescence and DIC images were acquired by toggling the microscope between the two modes while the camera was continuously collecting images at 32 fps. The displayed fluorescence images were 5-frame rolling averages to improve the signal-tonoise ratio

File Name: Supplementary Movie 5
Description: The transferrin-coated gold nanorod rotates, then stops, and finally starts rotation again. The regain of rotational and translational freedoms indicate that the nanorod-containing vesicle is cut off from the membrane.

File Name: Supplementary Movie 6
Description: This movie shows that the clathrin coat has disassembled from the transferrin-coated gold nanorod-containing vesicle after scission. The displayed fluorescence images were 5-frame rolling averages.

File Name: Supplementary Movie 7
Description: The transferrin-coated gold nanorod-containing vesicle is being actively transported inside the cell in DIC mode.

File Name: Supplementary Movie 8
Description: The transport of a transferrin-coated gold nanorod-containing vesicle in Parallax-DIC mode with 5D-SPT technique (Fig. 4).

File Name: Supplementary Movie 9
Description: Tracking of the transferrin-coated gold nanorod rotates on cell surface at the early stage of binding in Parallax-DIC mode with 5D-SPT technique (Supplementary Fig. 11A).

File Name: Supplementary Movie 10
Description: Tracking of the transferrin-coated gold nanorod went through CME and intracellular transport in Parallax-DIC mode with 5D-SPT technique (Supplementary Fig. 11C).

File Name: Supplementary Movie 11
Description: Intracellular transport of a transferrin-coated gold nanorodcontaining vesicle in Parallax-DIC mode with 5D-SPT technique (Supplementary Fig. 12).

File Name: Supplementary Movie 12
Description: Another example of intracellular transport of a transferrin-coated gold nanorod-containing vesicle in Parallax-DIC mode with 5D-SPT technique (Supplementary Fig. 13).
